# Status and content of outpatient preoperative education for rectal cancer patients undergoing stoma surgery provided by Japanese wound, ostomy, and continence nurses: a cross-sectional study

**DOI:** 10.1186/s12912-024-01857-5

**Published:** 2024-03-29

**Authors:** Yasumi Matsubara, Azusa Hirohata

**Affiliations:** 1 Kitasato University School of Nursing, 2-1-1, Kitazato, Minami-ku, Sagamihara, Kanagawa 252-0329 Japan; 2 Omiya Ichonaika Clinic, Kanasugi Nakamachi Bldg. 2F, 2-24-2 Nakamachi, Omiya-ku, Saitama-shi, Saitama, 330-0845 Japan

**Keywords:** Stoma, Rectal cancer, Preoperative education, Outpatient education, Stoma care, Wound, ostomy and continence nurse

## Abstract

**Background:**

Preoperative education can improve postoperative quality of life in patients undergoing stoma surgery. However, the prevalence and when, where, and how preoperative education is implemented are unclear. Therefore, this study aimed to assess the current status of outpatient preoperative education for patients undergoing stoma surgery for rectal cancer as perceived by nurses. Additionally, it sought to identify the information provided by Japanese healthcare providers as a part of preoperative education.

**Methods:**

This cross-sectional study included 1,716 wound, ostomy, and continence nurses (WOCNs) in charge of stoma clinics at Japanese hospitals. Unsigned self-administered survey forms were mailed to the participants, and paper- or web-based responses were obtained. The main questions included: overview of the participants and their facilities, provision of outpatient preoperative education, status of implementation, and preoperative education components. To examine the factors associated with preoperative education, the independent variable was the presence or absence of preoperative education in the clinic, and the explanatory variables were the years of experience as a nurse, years of experience as a WOCN, type of hospital, number of beds, and number of intestinal stoma surgeries per year. Regression analysis was performed.

**Results:**

We received 773 valid responses (valid response rate: 45%). Duration of experience as a nurse and as a WOCN were 24.6 and 10.9 years, respectively. Outpatient preoperative education was provided by 24% of the participants. Most preoperative education sessions were conducted for patients or caregivers. Preoperative education took 31–60 min per patient, and one to five patients received preoperative education each month. Booklets, ostomy appliances, and stoma models/dolls were used as supplementary materials for preoperative education. The most frequently mentioned components of preoperative education were stoma care, daily life, social security, stoma clinic, traveling and going out, quality of life after stoma surgery, and precautions for medical treatment. In addition, education on the use of restrooms on the go, disaster preparedness, defecation disorders after restorative proctectomy, and complications after stoma closure were considered necessary.

**Conclusions:**

The implementation rate of outpatient preoperative education was low (24%). Future challenges include the development of specific educational content and procedures suitable for the Japanese medical environment and the establishment of preoperative medical care teams for stoma surgery to promote the provision of outpatient preoperative education.

**Supplementary Information:**

The online version contains supplementary material available at 10.1186/s12912-024-01857-5.

## Background

Colorectal cancer is the most common cancer in Japan; the number of patients with this cancer type is approximately 150,000 in Japan, including approximately 50,000 patients with rectal cancer [1]. In recent years, advances have been made in the multidisciplinary treatment of rectal cancer with a combination of surgery and chemoradiation, and the 5-year relative survival rate for localized rectal cancer in Japan is as high as 95.7% [2].

However, defecation disorders, urination disorders, and sexual dysfunction can occur after rectal cancer surgery due to proctectomy and injury to the autonomic nerves. Rectal cancer surgeries can either be performed as temporary or permanent stoma surgeries, and changes in the excretory route, body image alteration, and stoma care can be burdensome for the patient [3, 4]. Patients are often highly anxious and have a poor quality of life during the period before and after stoma surgery [5, 6].

Stoma surgery is a predictable component of treatment; therefore, preoperative consultation and education are needed. In addition, stoma care requires close collaboration among physicians, nurses, and health and social care workers to provide technical, emotional, and social [7] support to the patients and guide treatment selection [8]. However, the shortened length of hospital stays and staff shortages in recent years have made it difficult to allocate the time needed to provide enough information to the patients. Many studies have reported that preoperative education in patients undergoing stoma surgery can improve postoperative quality of life [9, 10]. However, the prevalence and when, where, and how preoperative education is implemented are unclear.

## Methods

### Aim and design

The aim of this cross-sectional study was to evaluate the status of outpatient preoperative education for patients undergoing stoma surgery for rectal cancer as perceived by nurses. An additional objective was to identify specific educational content requirements in the Japanese healthcare context, which will aid the establishment of such clinics by serving as reference material.

### Participants and setting

We enrolled wound, ostomy, and continence nurses (WOCNs) in charge of stoma clinics in Japanese hospitals (1,716), regardless of age and sex; WOCNs in hospitals where rectal cancer surgery was not performed; WOCNs in hospitals without a stoma clinic; and WOCNs who were not in charge of a stoma clinic. Our inclusion criteria ensured that the participants had expert-level skills and knowledge in the care of stomas and defecation disorders and were involved in the direct care of patients with rectal cancer and the preoperative care of patients in outpatient settings and wards. We ensured this by selecting participants from the stoma clinic retrieval page of the Japanese Society of Wound, Ostomy, and Continence Management website [11] and the registrant list of certified nurses on the website of the Japanese Nursing Association [12].

### Data collection

Unsigned self-administered survey forms were mailed to participants, and paper- or web-based responses were obtained. Questionnaire items were then created independently, based on the findings of a study on preoperative education in patients undergoing stoma surgery [13]. The questionnaire used in our study was developed for this study (suppl. 1).

The main questions were as follows: years of experience as a nurse and as a WOCN, type of hospital, number of beds, and number of stoma surgeries per year (excluding surgeries on pediatric patients); the following items were used: provision of preoperative education in the clinic, status of preoperative education in the clinic, and preoperative education items considered necessary for patients undergoing stoma and rectal cancer surgeries. The data were collected from March 10 to June 20, 2022.

G-Power 3.1.9.7 [14] was used to calculate the ex-post sample size. The parameters used were as follows: medium effect size of f = 0.3, α error probability of 0.05, and power (1-β error probability) of 0.8 [15]. The total sample size required for the t-test was 771, and the non-centrality parameter for the chi-square test was 69.57.

### Data analysis

Descriptive statistics were calculated for the participants’ attributes, status of preoperative education in the clinic, and contents of the preoperative education. The group of nurses who had provided preoperative education in a clinic was compared to the group of those who had not done so. The mean number of years of nursing experience and experience as a WOCN were calculated and the differences between variables were analyzed using the Students’ t-test, the actual status of preoperative education in the clinic, and items considered necessary components of preoperative education. The mean numbers of hospitals of each type, beds, and intestinal stoma surgeries per year were analyzed using the chi-square test.

Regression analysis was conducted to identify factors associated with the presence or absence of preoperative education in the clinic. The independent variable was the presence or absence of preoperative education in the clinic, and the explanatory variables were the years of experience as a nurse, years of experience as a WOCN, type of hospital, number of beds, and number of intestinal stoma surgeries per year. Missing values were treated as variables with missing values were removed. Statistical analysis was performed using SPSS Version 28 (IBM Corp., Armonk, N.Y., USA), and statistical significance was set as *p* < 5%.

### Ethical considerations

The study was conducted in accordance with the principles of the Declaration of Helsinki (World Medical Association, 2001). Study procedures were reviewed and approved by the ethics committee of Kitasato University School of Nursing (approval number 2021-12-2, approval date: December 2, 2021). The study was conducted using a self-administered, anonymous questionnaire. Participants were notified in advance that no personal information would be collected, that submission of the questionnaire would be regarded as consent to participate in the study, and that consent could not be withdrawn after submission of the questionnaire.

## Results

### Participant characteristics

We received 773 valid responses (valid response rate: 45%). The mean number of years of experience as a nurse and as a WOCN were 24.6 (± 6.7) and 10.9 (± 5.2), respectively. 24% of the participants had provided preoperative education in a clinic. The number of beds and intestinal stoma surgeries were higher in the group of nurses who had provided preoperative education in a clinic than in the group of those who had not done so (*p* < 0.05). The number of intestinal stoma surgeries per year was higher in the group of nurses who had provided preoperative education in a clinic than in the group of those who had not done so (*p* < 0.05) (Table 1).

**Table 1:** Participant characteristics

			Preoperative education clinic		
		Total (*n* = 773)	Yes (*n* = 183)	No (*n* = 590)	*p* value
Years of experience as a nurse (± SD)		24.6 (± 6.7)	25.6 (± 7.2)	24.3 (± 6.6)	0.033*
Years of experience as a WOCN^1)^ (± SD)		10.9 (± 5.2)	12.9 (± 6.0)	10.3 (± 4.8)	< 0.001**
Type of hospital	University hospital	110 (14%)	34 (19%)	76 (13%)	0.013*
	General hospital	623 (81%)	133 (73%)	490 (83%)	
	Cancer hospital	26 (3%)	12 (6%)	14 (2%)	
	Other	2 ( 1%)	2 ( 1%)	6 (1%)	
	N/A	2 ( 1%)	2 ( 1%)	6 (1%)	
Number of beds	<200	102 (13%)	12 ( 6%)	90 (15%)	< 0.001**
	200 ~ 499	419 (54%)	78 (43%)	341 (58%)	
	500 ~ 999	210 (27%)	77 (42%)	133 (22%)	
	≧ 1000	37 (5%)	14 (8%)	23 (4%)	
	N/A	5 ( 1%)	2 ( 1%)	3 ( 1%)	
Number of stoma surgeries per year^2)^	0	44 ( 6%)	0 ( 0%)	44 ( 7%)	< 0.001**
	1 ~ 10	141 (18%)	22 (12%)	119 (20 $)	
	11 ~ 50	371 (48%)	81 (44%)	290 (49%)	
	51 ~ 99	159 (20%)	59 (32%)	100 (17%)	
	≧ 100	53 ( 7%)	20 (11%)	33 ( 6%)	
	N/A	5 ( 1%)	1 ( 1%)	4 ( 1%)	

^1)^Wound, Ostomy and Continence Nurse; ^2)^ intestinal stoma (without pediatric)

t-test; chi-square test, *: *p* < 0.05, **: *p* < 0.01

### Status of outpatient preoperative education

#### Overview of outpatient preoperative education

Most patients (96%) or family members (95%) were eligible for preoperative education in the clinics. Preoperative education took 31–60 min per patient (78%). One to five patients per month received preoperative education (81%), and preoperative education was performed in stoma clinics (79%) (Table 2).

**Table 2:** Overview of preoperative education clinic

Item		(%) n = 183
Subject	Patient	176 (96%)
	Caregiver/family	174 (95%)
	Others	13 (7%)
Time (min)	≧ 30	30 (16%)
	31–60	143 (78%)
	6190	7 (4%)
	N/A	3 (2%)
Number of times per month	1–5	148 (81%)
	6–10	20 (11%)
	11–15	4 (2%)
	≧ 16	4 (2%)
	N/A	7 (4%)
Place	Stoma clinic	144 (79%)
	Outpatient clinic	73 (40%)
	Outpatient waiting room	4 (2%)
	Others	25 (14%)
Reservation system	Yes	61 (33%)
	No	122 (67%)
Timing	Preoperative examination date	93 (51%)
	After determination of hospitalization date	90 (49%)
	Date of disease notification	46 (25%)
	Date of the first visit	18 (10%)
	Others	79 (43%)

#### Supplementary materials used for outpatient preoperative education

**Fig. 1:**
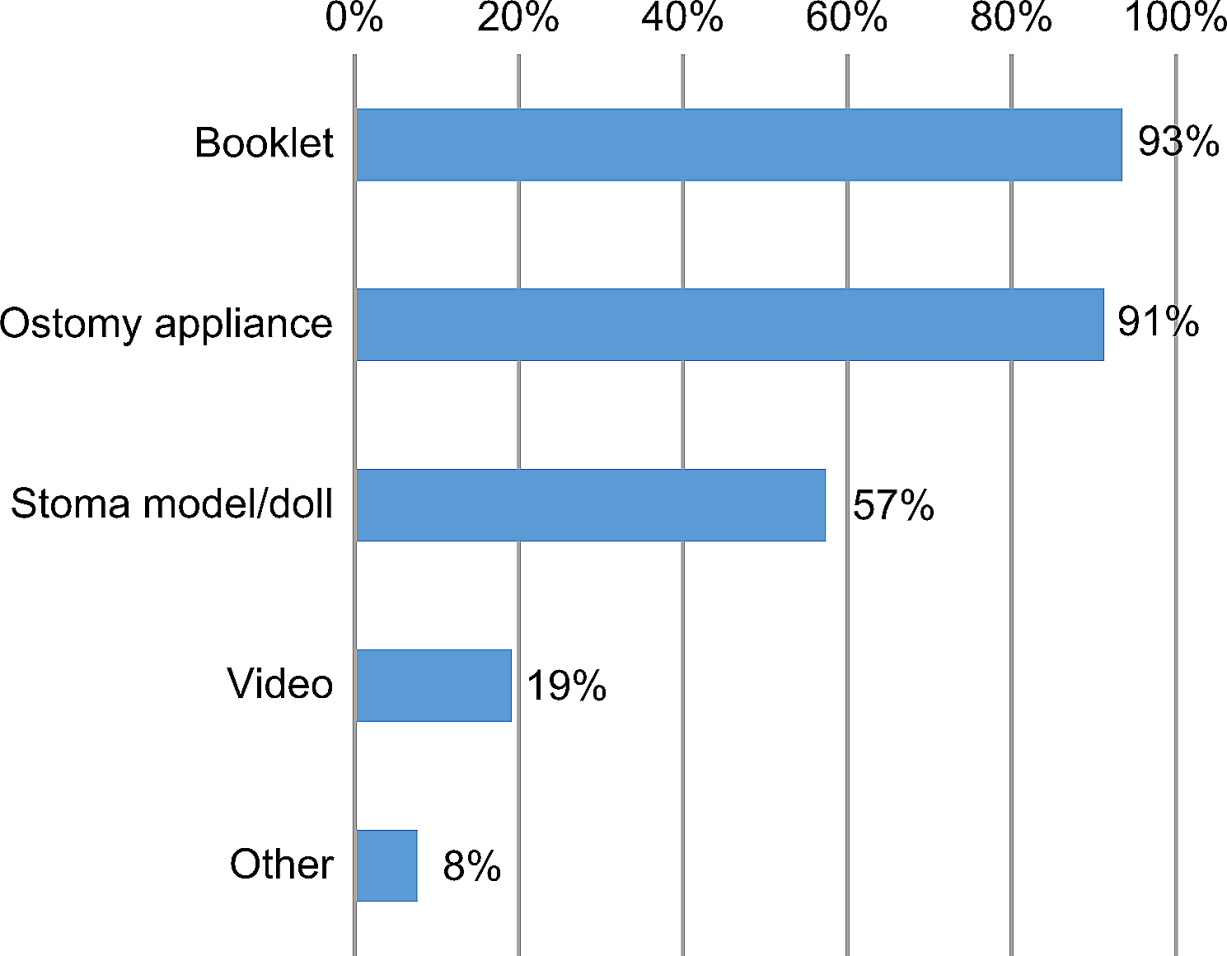
Supplementary materials used for preoperative education in the clinics

The most commonly used supplementary materials were booklets (93%) and ostomy appliances (91%), followed by stoma models and dolls (57%), and videos (19%) (Fig. 1).

### Factors related to whether outpatient preoperative education is provided

Logistic regression analysis revealed that the presence of preoperative education in the clinic was significantly associated with the years of experience as a WOCN, number of beds, and number of stoma surgeries per year (*p* < 0.05) (Table 3).

**Table 3:** Factors related to a preoperative education clinic

	Odds ratio	95% CI		*p* value
		Lower limit	Upper limit	
Years of experience as a nurse	1.024	0.99	1.06	0.173
Years of experience as a WOCN^1)^	0.909	0.869	0.95	< 0.001**
Type of hospital	0.732	0.509	1.053	0.092
Number of beds	0.701	0.519	0.948	0.021*
Number of stoma surgeries per year^2)^	0.708	0.561	0.893	0.004**

logistic regression analysis, ^*^: *p* < 0.05, ^**^: *p* < 0.01

^1)^Wound, ostomy, and continence nurse; ^2)^ intestinal stoma (without pediatric)

### Preoperative education content considered necessary

**Fig. 2:**
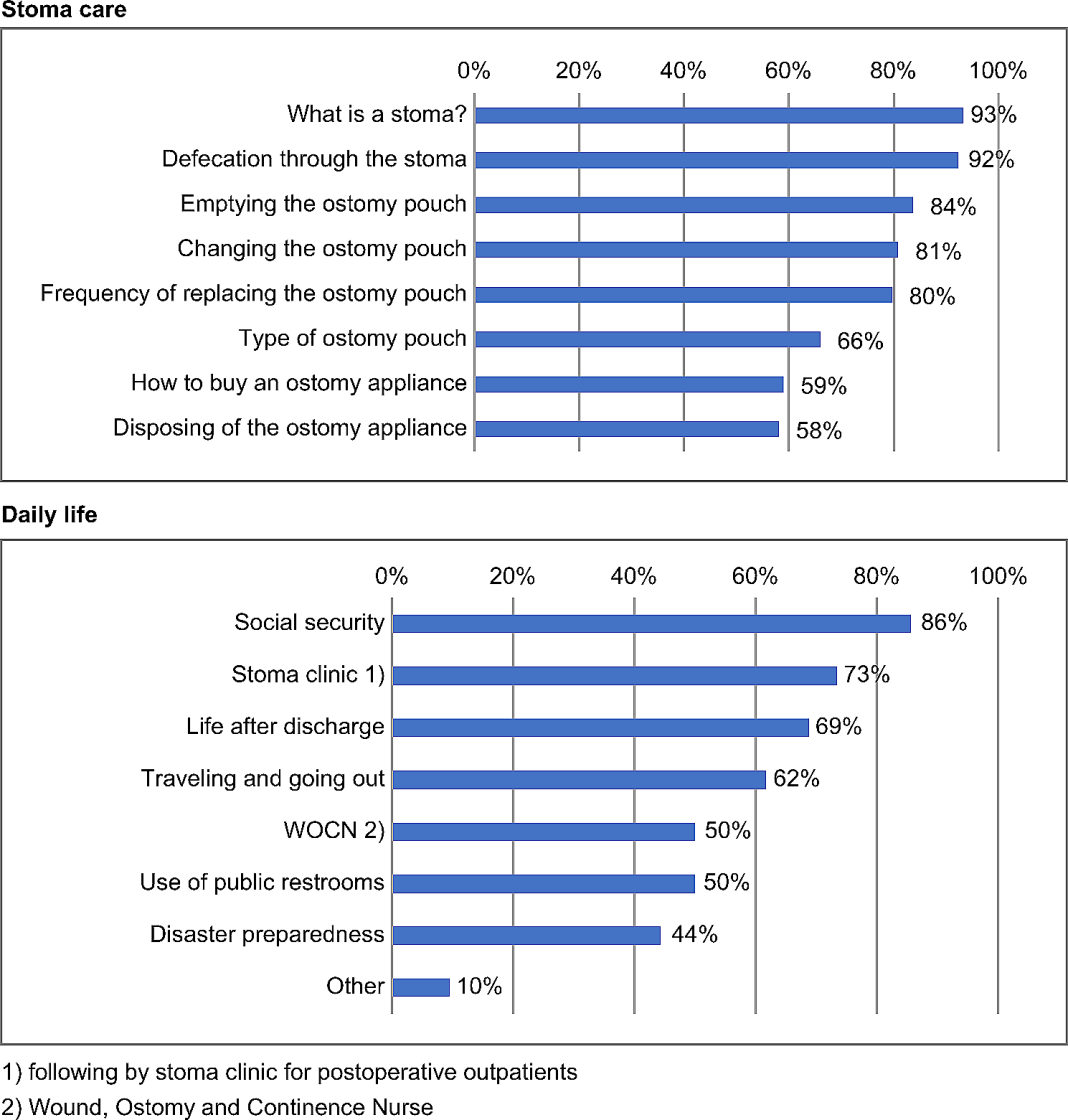
Preoperative education provided to patients undergoing stoma surgery

In terms of educational content related to stoma care, “what is a stoma?” (93%) and “defecation through the stoma” (92%), followed by “emptying the ostomy pouch” (84%), and “changing the ostomy pouch.” In terms of educational content related to daily life, social security (86%) was the most common response, followed by stoma clinic for postoperative outpatients (73%), life after discharge (69%), and traveling and going out (62%) (Fig. 2).

**Fig. 3:**
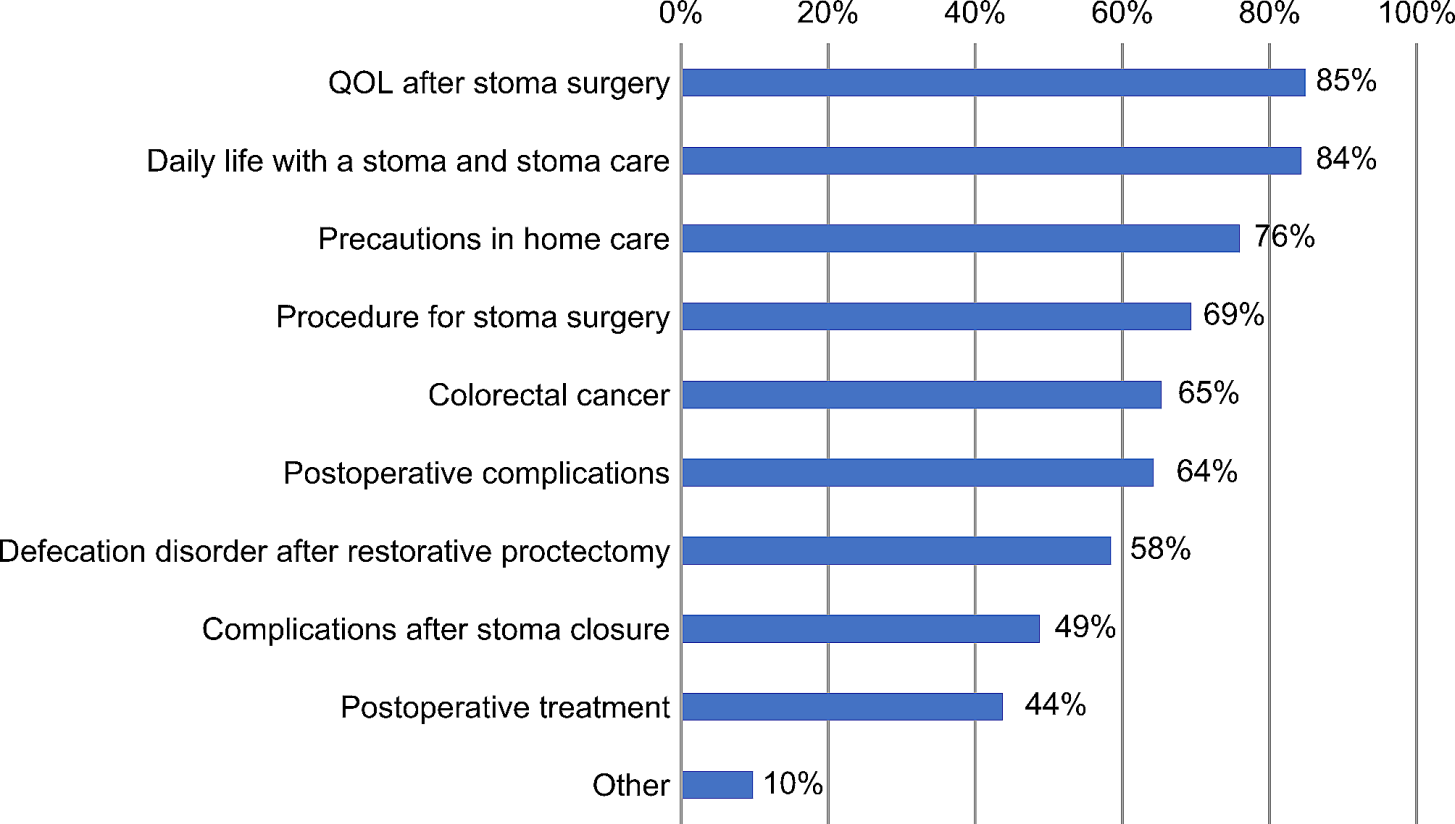
Preoperative education provided to patients with rectal cancer

In terms of educational content related to rectal cancer surgery, quality of life after stoma surgery (85%) and daily life with a stoma and stoma care (85%) were the most common, followed by precautions in-home care (76%), and procedure for stoma surgery (69%) (Fig. 3).

## Discussion

The usefulness of preoperative education for patients undergoing stoma surgery has been demonstrated in previous studies [16–24]. Education by stoma specialist nurses has been reported to improve patient quality of life [9, 10] and reduce anxiety [22, 23], and ongoing education and counseling before surgery facilitate adaptation to life with a stoma [9]. Outpatient preoperative education by medical care teams has also been shown to significantly accelerate the initiation of postoperative self-care [13, 22]. Preoperative education has also been shown to reduce length of hospital stays [22, 24] and reduced ostomy-related complications [22].

Our study revealed that the percentage of patients receiving outpatient preoperative education was low (24%). The low prevalence of this education in Japan can be attributed to the fact that preoperative education for stoma surgery is not covered by health insurance and to a lack of medical personnel. Additionally, WOCNs may find it difficult to allocate sufficient time for providing preoperative education on stoma care to each patient. It is difficult to secure sufficient time to provide stoma-related information because of the short length of hospital stays and insufficient staff [21].

According to a national survey of WOCNs in Japan, 87.6% of WOCNs worked in hospitals [25]. In Japan, many WOCNs are employed in dedicated or full-time roles to manage pressure ulcers, and they charge medical fees, which generate revenue for hospitals. Outpatient preoperative education is designed to enable early discharge and provide physical and emotional support to patients in collaboration with multiple professionals, depending on individual patient risk factors and conditions. For WOCNs to have time for outpatient preoperative education, it is necessary that these charges be covered by health insurance and establish a system of team-based collaborative medical care.

Our survey showed that education on the use of restrooms and disaster preparedness was provided by approximately 50% of the participants. According to a nationwide survey of ostomates, 41.5% of respondents reported having problems in their daily lives, such as problems in the supply of ostomy appliances and self-management in case of a disaster [26]. It is important to provide preoperative information about disasters that may occur anywhere and at any time.

However, it may be difficult for patients to understand if a large amount of information is provided in outpatient preoperative education. Additionally, there are individual differences in patients’ lives, information needs, and literacy. Therefore, to promote patient quality of life, an ongoing care plan involving the patient and family is necessary, starting before surgery [27].

Our study showed that videos were used as supplementary materials in only 19% of the preoperative education sessions. Therefore, in addition to face-to-face preoperative education provided by WOCNs, the use of videos, which can be viewed at any time and place, as supplementary materials, may help deepen the patients’ understanding.

The years of experience as a WOCN, number of beds, and number of intestinal stoma surgeries per year were significantly related to whether preoperative education was provided in the clinic. Preoperative education was more commonly provided on an outpatient basis with experienced WOCNs with exceptional practice skills than in clinics without such WOCNs.

Previous studies have reported that the preoperative involvement of specialized stoma nurses leads to shorter postoperative hospital stays and cost-effectiveness [28].

One limitation of this study is that the survey was conducted during the coronavirus disease pandemic in Japan, which may have affected the response rate. In addition, the survey was conducted among WOCNs whose information was publicly available on the web, and it is possible that multiple WOCNs at the same facility responded to the survey. The fact that the study included only Japanese nurses limits the generalizability of the present findings because of healthcare system variations and cultural specificity.

However, the survey revealed a low prevalence of outpatient preoperative education in Japan, suggesting future challenges. In addition, the findings regarding the educational content considered necessary may inspire new ideas for the diffusion of outpatient preoperative education that is appropriate for the Japanese medical environment.

## Conclusions

The percentage of patients with rectal cancer scheduled for stoma surgery who received outpatient preoperative education was low (24%). The most common topics on which preoperative education was provided were stoma care, daily life, social security, stoma clinics, traveling and going out, quality of life after stoma surgery, and precautions during medical treatment. In addition, education on the use of restrooms on the go, disaster preparedness, defecation disorders after restorative proctectomy, and complications after stoma closure were considered necessary. Future challenges include developing specific educational content and procedures appropriate for the Japanese medical environment and establishing preoperative medical care teams for stoma surgery.

### Electronic supplementary material

Below is the link to the electronic supplementary material.

Supplementary Material 1

## Data Availability

The datasets used and/or analyzed during the current study are available from the corresponding author upon reasonable request.

## Abbreviations

WOCN: Wound, ostomy and continence nurse
