# Supplementary material for: Status and content of outpatient preoperative education for rectal cancer patients undergoing stoma surgery provided by Japanese wound, ostomy, and continence nurses: a cross-sectional study
Source: BMC Nurs. 2024 Mar 29;23:218. doi: 10.1186/s12912-024-01857-5 (PMC10979554; doi:10.1186/s12912-024-01857-5)
Supplement: Supplementary file 1 — Supplementary Material 1 [file 12912_2024_1857_MOESM1_ESM.docx]

**Survey of Preoperative Education for Patients Undergoing Stoma Surgery**

**for rectal cancer patients**

If you would like to participate in the study, please write ☑ in the box below.

- I have been informed about the " Survey of Preoperative Education for Patients Undergoing Stoma Surgery for rectal cancer patients " and will participate in the study. I also understand that submitting the questionnaire with my answers will be considered as my consent to participate in the study and that I cannot withdraw my consent after submitting my answers because this survey is anonymous, and individuals cannot be identified.

**Ⅰ.We would like to ask you a few questions about yourself and your institution.**

| 1. Please check (☑) the type of organisation you belong to. | | | |
| --- | --- | --- | --- |
| □ | 1) University hospital | □ | 2) General hospital |
| □ | 3) Cancer hospital | □ | 4) Others |
| 2. Please check (☑) the number of beds in your facility that apply. | | | |
| □ | 1) Less than 200 beds | □ | 2) 200-499 beds |
| □ | 3) 500-999 beds | □ | 4) More than 1000 beds |
| 3. Please enter the number of years of nursing experience as a nurse in parentheses. ( ) Years | | | |
| 4. Please enter the number of years of experience as a WOCN in parentheses. ( ) Years | | | |

**Ⅱ.We ask about the number of ostomies performed and whether there are clinics that specialize in preoperative ostomy education (hereafter referred to as preoperative education clinics).**

| 1. Please indicate the number of gastrointestinal stomas (excluding pediatric) installed during the period January through December 2021. | | | |
| --- | --- | --- | --- |
| □ | 1) 1-0 cases | □ | 2) 1-10 cases |
| □ | 3) 11-50 cases | □ | 4) 51-99 cases |
| □ | 5) More than 100 cases |  |  |
| 1. Do you offer ambulatory preoperative education? | | | |
| □ | 1) Yes | □ | 2) No |
| 3.Is it by reservation system? | | | |
| □ | 1) Yes | □ | 2) No |

**Ⅲ.If you answered "yes" to Ⅱ(2), we would like to ask you about the status of your preoperative educational outpatient clinic.**

| 1. Approximately how much time do you spend on each case? (Convert one case if a family member was present with the patient and one case if only a family member was involved.) | | | |
| --- | --- | --- | --- |
| □ | 1) Less than 30 minutes | □ | 2) 31-60 minutes |
| □ | 3) 61-90 minutes | □ | 4) More than 91 minutes |
| 1. What is the average number of preoperative educational outpatient visits per month? (Convert one case if a family member was present with the patient and one case if only a family member was present.) | | | |
| □ | 1) 1-5 cases | □ | 2) 6-10 cases |
| □ | 3) 11-15 cases | □ | 4) More than 16 cases |
| 3.When is preoperative education provided? | | | |
| □ | 1) Preoperative examination date | □ | 2) After determination of hospitalization date |
| □ | 3) Date of disease notification | □ | 4) Date of the first visit |
| □ | 5) Others |  |  |
| 4.Where will the preoperative ostomy education take place? | | | |
| □ | 1. Stoma clinic | □ | 2) Outpatient clinic |
| □ | 3) Outpatient waiting room | □ | 4) Others |
| 5.Who is preoperative education provided for? | | | |
| □ | 1. Patient | □ | 2) Caregiver/family |
| □ | 3) Others |  |  |
| 6.Please check all that apply to the additional information you use to educate patients in the preoperative education clinic. | | | |
| □ | 1) Self-made booklets | □ | 2) Self-made videos |
| □ | 3) Manufacturer-produced booklet | □ | 4) Manufacturer-produced video |
| □ | 5) Ostomy appliance | □ | 6) Stoma model / doll |
| □ | 7) Internet videos | □ | 8) Other |

**Ⅳ Please check (☑) all that apply to your preoperative rectal cancer education needs.**

| □ | 1) About colorectal cancer | □ | 2) Procedure for stoma surgery |
| --- | --- | --- | --- |
| □ | 3) Methods of stoma surgery | □ | 4) Complications after surgery |
| □ | 5) Quality of life after stoma surgery | □ | 6) Complications after stoma closure |
| □ | 7) Followed by precautions in-home care | □ | 8) Fecal incontinence after anal preservation surgery |
| □ | 9) Postoperative treatment | □ | 10) Daily life with a stoma and stoma care |
| □ | 11) Other | | |

**Ⅴ Please check all that apply to your preoperative ostomy education needs.**

| □ | 1) What is a stoma | □ | 2) How to defecate through the stoma |
| --- | --- | --- | --- |
| □ | 3) How to empty the ostomy pouch | □ | 4) How to change the ostomy pouch |
| □ | 5) Frequency of changing ostomy pouch | □ | 6) Disposal of used ostomy pouch |
| □ | 7) Types of ostomy pouch | □ | 8) Followed by stoma clinic for postoperative outpatients |
| □ | 9) What is a WOCN | □ | 10) How to buy a ostomy appliance |
| □ | 11) Social Security | □ | 12) Life after discharge |
| □ | 13) How to go out and travel | □ | 14) How to use the toilet on the go |
| □ | 15) Disaster preparedness | □ | 16) Other |

That's all for the survey. Thank you for your cooperation.
